# The brain lateralization and development of math functions: progress since Sperry, 1974

**DOI:** 10.3389/fnhum.2023.1288154

**Published:** 2023-10-27

**Authors:** Elena Salillas, Silvia Benavides-Varela, Carlo Semenza

**Affiliations:** ^1^Department of Psychology and Sociology, University of Zaragoza, Zaragoza, Spain; ^2^Department of Developmental Psychology and Socialisation, University of Padova, Padua, Italy; ^3^Padova Neuroscience Center, University of Padova, Padua, Italy

**Keywords:** brain lateralization, mathematical cognition, neuroimaging, lesion studies, adults, numerical development

## Abstract

In 1974, Roger Sperry, based on his seminal studies on the split-brain condition, concluded that math was almost exclusively sustained by the language dominant left hemisphere. The right hemisphere could perform additions up to sums less than 20, the only exception to a complete left hemisphere dominance. Studies on lateralized focal lesions came to a similar conclusion, except for written complex calculation, where spatial abilities are needed to display digits in the right location according to the specific requirements of calculation procedures. Fifty years later, the contribution of new theoretical and instrumental tools lead to a much more complex picture, whereby, while left hemisphere dominance for math in the right-handed is confirmed for most functions, several math related tasks seem to be carried out in the right hemisphere. The developmental trajectory in the lateralization of math functions has also been clarified. This corpus of knowledge is reviewed here. The right hemisphere does not simply offer its support when calculation requires generic space processing, but its role can be very specific. For example, the right parietal lobe seems to store the operation-specific spatial layout required for complex arithmetical procedures and areas like the right insula are necessary in parsing complex numbers containing zero. Evidence is found for a complex orchestration between the two hemispheres even for simple tasks: each hemisphere has its specific role, concurring to the correct result. As for development, data point to right dominance for basic numerical processes. The picture that emerges at school age is a bilateral pattern with a significantly greater involvement of the right-hemisphere, particularly in non-symbolic tasks. The intraparietal sulcus shows a left hemisphere preponderance in response to symbolic stimuli at this age.

## Introduction

Roger Sperry and colleagues, in their seminal article on the split-brain condition (Sperry et al., 1969) concluded that calculation must be considered, along with speech and writing, a left hemisphere function and that the contribution of the right hemisphere to calculation was almost nil. In fact, they wrote, “tests for mathematical performance in the minor hemisphere with nonverbal readout and with the sensory input restricted to the left visual field or the left hand, indicate … that the capacity for calculation on the minor side is almost negligible. By manipulating marbles or dowel sticks, watching spots of light flashed to the left field and pointing with the left hand, … (split brain) patients may succeed in matching numbers or in adding one to numbers below 10, but they fail when required to add or subtract two or higher numbers and they fail also at the simplest tasks in multiplication and division.” However, Sperry (1974) later observed how the right hemisphere could, in fact, perform additions up to sums less than 20, but that was the only exception to a complete left hemisphere dominance.

Sperry’s conclusions were consistent with traditional wisdom. His contemporaries, in fact, considering the outcome of lateralized focal lesions, all agreed that primary acalculia, the authentic deficit in arithmetic, was found only after left hemisphere lesions, while a right hemisphere lesion could only lead to calculation problems secondary to visuospatial disorders. Observed acalculia was thus classified in three main categories (Berger, 1926; Hécaen et al., 1961; Hécaen and Angelergues, 1961): (1) “primary acalculia” (“anarithmethia”), the disturbance of core math functions, resulting from lesions of the left parietal areas; (2) secondary acalculia, determined by language problems (mostly reading and writing disorders), resulting from lesions in left perisylvian, language-related, areas, and (3) secondary, “spatial” acalculia, following lesions of the posterior areas of the right hemisphere, related to spatial disorders, like, for instance, unilateral spatial neglect. In this classification, therefore, there was no primary role for the right hemisphere.

Fifty years after Sperry’s article, the notions about brain laterality of calculation are radically changed. The contribution of new theoretical and instrumental tools leads to a much more complex picture, whereby, while a left hemisphere dominance for math in the right-handed is confirmed for most functions, several math related tasks seem to be carried out primarily in the right hemisphere. Moreover, even simple calculation, it is recognized, is sustained by complex networks where each of the two hemispheres may have a role of its own. Importantly, the developmental trajectory in the brain lateralization of math functions has also been clarified.

The aim of this article is to summarize the state of the art about the lateralization of math functions, including its development under the influence of growth and education. In this last respect, existent meta-analyses will be scrutinized in terms of lateralization. The sources of evidence are lesion cases and neuroimaging techniques in adults as well as in developmental populations.

## Lesion cases

Focal brain lesions may provoke so called “acalculia.” As mentioned in the introduction, since the earliest observations, a tripartite classification was proposed. Rather than being an exclusive result of a right or a left lateralized lesion, however, these different types of acalculia have a different frequency in left and in right hemisphere lesions (Hécaen et al., 1961; Hécaen and Angelergues, 1961; Hécaen, 1962). Although different proportions are reported in various studies (see the review by Semenza, Benavides-Varela and Salillas, in press), anarithmethia and alexia/agraphia were always predominant after left hemisphere lesions and spatial acalculia was found predominant in right hemisphere lesions. In spatial acalculia patients are unable to respect the order and the position of digits in relation to each other; when the cause is neglect, one side of the calculation space is ignored. Notably, in the earlier literature, simple mental calculation, like tables, in which the possibility that errors originate from a spatial disorder is very unlikely, was occasionally found to be affected also after right hemisphere lesions (Hécaen, 1972). The nature of such errors, however, was never discussed.

Warrington and James (1967) addressed the issue of lateralization using very basic numerical tasks. They compared left and right brain damaged patients on tachistoscopic estimation of number of dots with untimed counting of dots. This last task did not show lateralization effects, while the estimation of number of dots was worse in the right parietal patients, as a consequence of a disturbance of approximation abilities. Dehaene and Cohen (1991, 1997) later appealed to this hypothesis in reporting three cases of acalculia who featured a double dissociation between calculation and approximation abilities. Importantly, they proposed that simple single digit calculation can be solved either via a “direct” route or via an “indirect,” semantic route. The direct route (mainly used for addition and multiplication and sustained by a left-sided cortico-subcortical circuit) would elicit the rote memory for the operation. The indirect route (preferentially used in subtraction and located bilaterally in the inferior parietal cortex and in the left perisylvian network) would monitor the plausibility of a result retrieved by the direct route by referring to approximate magnitude knowledge.

The anatomical correlates of the main acalculia types were the object of several group studies conducted via extensive batteries of math tasks. Grafman et al. (1982) compared left anterior, left posterior, right anterior, and right posterior patients on written additions, subtractions, multiplications, and divisions, including multidigit ones. The worst performance was found in posterior groups, the left one being by far the most affected. This result still held when the influence of impairments of intelligence, visuospatial disorders, and aphasia was considered. No laterality effect was shown in the quality of errors. Similar results were found by Rosselli and Ardila (1989) in a more comprehensive study, including number reading and writing, number transcoding from Arabic code to alphabetical code and vice versa, number size judgements, reading of arithmetical signs, mental and written calculation, successive additions, and subtractions, aligning of numbers for complex additions, and orally presented numerical problems. The left posterior group committed the highest number of errors, the quality of which seemed to vary according to the concomitant aphasia category; the right anterior and posterior groups did not differ from each other. Errors in the right hemisphere groups were analyzed in a later study (Ardila and Rosselli, 1994).They appeared mostly in written calculation, where however the strategies and sequencies required to add, subtract, multiply and divide were preserved; moreover, arithmetical signs were correctly interpreted. In reading and writing numbers, the same spatial difficulties observed in reading and writing in general were observed. Disturbances of arithmetic reasoning and in simple calculation were also found. The authors attributed even these last errors to a spatial deficit. They argued, without further explanation, that they were related to the “general loss of automatisms” reported by Luria (1966).

Langdon and Warrington (1997) interestingly contrasted right and left lesions in calculation and arithmetical reasoning. They found that, unlike for calculation, the right hemisphere is involved in arithmetical reasoning as much as the left hemisphere. In Basso et al. (2000) only a small proportion of right hemisphere patients were found to be affected by acalculia if not affected by neglect: no report on the nature of their errors is provided. In their left hemisphere group with aphasia, almost half of the patients did not show signs of acalculia. The proportion of aphasic patients without acalculia was reported to be about a quarter of the group studied in Delazer et al. (1999), where specific failures in specific math functions were related to specific aphasic syndromes. Thus, multiplication was especially hard for Broca’s aphasics, while calculation procedures were mostly affected in Wernicke’s aphasia. Confirming an earlier finding by Seron and Deloche (1983), syntactic errors (substitution of multipliers, e.g., 4,000 instead of 400) were more frequent in Broca’s aphasia, whereas lexical errors (substitution of digits within position, e.g., 103 instead of 102) were mainly observed in Wernicke’s aphasia.

More recent studies investigated the specific role of the right hemisphere more directly. A single case reported by Granà et al. (2006) provided evidence for a previously unknown specific function of the right hemisphere. The patient’s lesion was limited to the posterior areas of the right hemisphere. The only domain where he committed errors was that of procedures in multidigit written multiplication. In contrast, he was able to describe the procedures in words and knew all the steps necessary to perform the operation. His errors were not determined by a generic inability to deal with spatial material, or deficits like neglect. For example, he added the carry to the leftmost side of the multiplicand, or of the partial product, or to the partial product in the row below. He seemed to know *when* and *how* the carry must be added but he clearly forgot *where*. The authors concluded that he was lacking knowledge of the visuo-spatial layout representation specific to multiplication, that is learned at school and guides written multiplication.

Reading aloud and writing on dictation multidigit numbers containing zero has also been found to be a right hemisphere specific function (Benavides-Varela et al., 2016). Surprisingly, right hemisphere patients, who are free from language disorders, committed many errors in these tasks, and not in numbers that do not contain zero. No relation was found of such errors with neglect or with generic cognitive impairment. These errors seem to reveal the existence of a basic mechanism entailing parsing and the ability to set-up empty-slot structures required for processing *zeros* in complex numbers. As also confirmed in a subsequent study on a huge group of stroke patients (Haupt et al., 2017), this ability can be located in the right insula and its surroundings.

Benavides-Varela et al. (2014) further investigated the influence of neglect on calculation deficits. Right hemisphere patients without neglect still showed an impairment of math. Importantly, these patients failed in one-digit mental subtraction and multiplication, tasks that do not require written visuospatial abilities. Thus, the authors concluded, patients with unilateral right hemisphere lesions did not just ignore the spatial arrangement of multi-digit written operations but were affected by specific representational deficits in simple mental calculation. Similar conclusions were reached by Dormal et al. (2014): neglect patients performed worse in subtraction, while they performed well on matched additions. According to the authors this result demonstrated a causal relationship between attending to the left side of space and solving subtractions.

Benavides-Varela et al. (2017) studied right hemisphere acalculia by administering NADL, a battery for numerical abilities (Semenza et al., 2014). Impairments were found in number comprehension, transcoding, and written operations. Interestingly, pure arithmetical errors (failure with number facts, carrying, borrowing, etc.) were more numerous than errors of spatial origin and were associated with lower parietal lesions; they were not predicted by visuo-spatial abilities. In contrast, spatial errors (misalignment, omissions of the left-most digits, number inversions, etc., often accompanied by neglect) were associated with lesions in fronto-temporoparietal areas; they were predicted by composite measures of visuo-spatial attentional and representational abilities.

The respective role of the two hemispheres in the retrieval of arithmetical facts and other simple operations was clarified using direct cortical electrostimulation (DCE) during tumor neurosurgery. Stimulation of the right parietal lobe impaired simple subtraction (Yu et al., 2011). Later studies identified positive sites in the right hemisphere also in addition, in the supramarginal gyrus, and in multiplication, in the angular gyrus, the supramarginal gyrus, the interparietal sulcus and the superior parietal lobule (Della Puppa et al., 2013, 2015). Crucial information came from the analysis of substitution errors (omissions were less than 5%; Semenza et al., 2017). Disruption on right parietal areas provoked multiplication errors, mostly consisting in replacing the correct solution with another table solution (e.g., 8×3 = 32 or 8 × 3 = 16). The authors attributed this effect to the left hemisphere, where the retrieval of tables is supported, taking over the operation. In contrast, disruption of the left hemisphere resulted in approximation errors (e.g., 8 × 3 = 25). This would be due to the activity of the right hemisphere, where approximation takes place. No individual site was positive for both addition and multiplication. Errors therefore do not simply result from shortage of processing resources, which would have not provoked operation-specific deficits. Stimulation on the left hemisphere during simple addition led to approximation errors. Underestimation of the correct result was found after inhibition in both hemispheres: however, a larger deviation from correct was found after right hemisphere inhibition, reflecting a deficit in the approximation mechanism.

## Neuroimaging studies of numerical processing in adults

Studies on lateralization conducted via neuroimaging have been mostly inspired by the Triple Code Model (TCM, Dehaene, 1992; Dehaene and Cohen, 1995), a theoretical framework encompassing three main numerical representations and the corresponding brain areas.

The first proposed numerical representation is the *Analogic Magnitude Representation* (*Analogical Code*), a pre-verbal representation, common to non-human species and pre-verbal infants, involved in virtually all numerical processes. It carries the numerical meaning, a “mental number line” where numerosity is represented in a compressed manner, according to Weber’s law. It allows for the approximate contrast between sets of objects differing in numerosity. An approximate numerosity estimation system (*Approximate Number System* – ANS) is referred to in some studies (e.g., Piazza, 2010).

This analogical code is located (according to a meta-analysis of neuroimaging data, Dehaene et al., 2003) bilaterally in the horizontal intraparietal sulcus (hIPS). Further studies confirmed this location using adaptation paradigms (e.g., Piazza et al., 2004, 2007; Cantlon et al., 2006; Cohen Kadosh et al., 2007; Kadosh et al., 2011) and comparison tasks (e.g., Pinel et al., 1999, 2001) where the ratio, or the distance, between numerosities is computed. A higher reliance on the right parietal areas for analogue quantities has also been proposed (Faye et al., 2019). Cohen Kadosh et al. (2007) proposed that while the left parietal areas might process magnitude independently of format, the right parietal areas would process magnitude in a format dependent manner.

The pattern of lateralization, is believed, may change when symbols are attached to numerical meanings. Vogel et al. (2015) showed hemispheric differences in the IPS using symbolic vs. non-symbolic stimuli. In a recent meta-analysis by Escobar-Magariño et al. (2022) lateralization indexes suggested a prevalent left IPS dominance when the stimuli were symbolic and a right IPS dominance when the stimuli where non-symbolic.

A second numerical representation proposed by the TCM is the *Visual Arabic Form* (or *Arabic Code*), which works in the recognition of Arabic digits. It is located in the occipito-temporal cortices. The specific site of the so-called Number Form Area (NFA) has been the object of several studies in contrast to the visual word form area (VWFA). Electrocorticography studies (Shum et al., 2013) seem to suggest a bilateral location. However, Hannagan et al. (2015) showed an asymmetry in favor of the right inferior temporal gyrus, and Yeo et al. (2017) meta-analysis indicated the right inferior temporal cortex as specific for the NFA. An Arabic number, unlike letters or letter strings, carries indeed numerical semantic information: this fact makes it likely a wider bilateral location of this recognition system. A complex network would be at work, encompassing the parietal regions bilaterally, and right lateralized superior and inferior frontal regions. Hannagan et al. (2015) suggested that the left NFA could be less differentiated from the VWFA, and less specific to number symbols. Nemmi et al. (2018) suggested that the right predominance of NFA could be explained by the default functional connectivity between inferior temporal gyrus (ITG) and IPS in the right hemisphere before symbols are learned. The connectivity with left parietal areas, they propose, would be established later, with the acquisition of symbols.

Finally, the *Verbal Word Frame* (*Auditory-Verbal Code*) organizes numbers as word sequences, supporting counting. Importantly, it sustains the retrieval of arithmetic facts, which is achieved phonologically through rote verbal memory. Consistently with its verbal nature, it is lateralized (Burbaud et al., 1995; Dehaene et al., 1999) in the left perisylvian areas and, specifically, in the left angular gyrus (AG). A training study of Delazer et al. (2003) showed a shift from IPS to left AG when learning of new arithmetic facts is achieved. This reflected a shift from quantity-based processes in IPS, to automatic retrieval of arithmetic facts in left AG. Additional studies converged with these results with the addition of the left hippocampus (e.g., Delazer et al., 2005; Ischebeck et al., 2006; Grabner et al., 2009; Bloechle et al., 2016).

The TCM still constitutes a valid framework for the location of math functions. However, after over a quarter of a century, it needs additions. In particular, new important neuroimaging and electrophysiological findings have clarified the role of the right hemisphere, in keeping with the abovementioned research in the clinical domain. A meta-analysis by Arsalidou and Taylor (2011) evidenced how parietal areas are bilaterally relevant for addition, subtraction and multiplication. While addition showed left hemisphere dominance, subtraction appears to be sustained bilaterally and a right hemisphere dominance was found for multiplication (this last finding, surprising in consideration of a vast literature), was nonetheless confirmed in Rosenberg-Lee et al. (2011b).

The specific role of each hemisphere in simple calculation has been investigated by means of Magnetoencephalography (MEG). As stated by TCM, and in keeping with DCE findings (Semenza et al., 2017), table retrieval seems to rely on a left frontoparietal circuit, while an analogous right frontoparietal circuit appears to support approximation processes during calculation (Arcara et al., 2021). Moreover, in Salillas et al. (2021) the verification of incorrect multiplication solutions related to the operands (3 × 4 = 16) required a left hemisphere network, while the verification of multiplication solutions unrelated to the operands (3 × 4 = 13) needed a right hemisphere network. In the first case there is an interference of stored memories for arithmetic facts that is absent in the second case, hence the difference. Connectivity measures showed evidence for a loop between the left dorsolateral areas and the left AG, which might play a crucial role in resolving the interference of incorrect, yet related solutions. Inferior frontal areas, bilaterally, are probably used for final selection. Consistently with these findings Arcara et al. (2021) showed how retrieval of easy items (fastest responses) need the left AG in a first stage. The right dorsolateral prefrontal cortex as well right parietal areas, later contribute to identifying the correct solution.

### The lateralization of the mental number line

One important aspect of numerical cognition addressed with only a few neuroimaging studies is that of the lateralization of the “mental number line,” a representation whereby, in left to right readers, small numbers are on the left and large numbers are on the right. The best demonstration of such representation is the so called SNARC (Spatial Numerical Association of Response Codes, Dehaene et al., 1993) effect: within a given interval, small numbers are responded faster with the left hand and larger numbers are responded faster with the right hand. Using Functional Near-Infrared Spectroscopy (fNIRS), Cutini et al. (2014) applied a comparison task and contrasted right and left responses to small and large numbers. Hence, they could contrast the hemodynamic response to SNARC compatible responses vs. SNARC incompatible responses. Bilateral HIPS and left AG appeared related to the SNARC effect and hIPS overlapped with the response to distance between numbers. The distance effect is a marker of the access to magnitude. Thus, these authors suggested a close link between space and the core number process during numerical comparison. Another recent study addressed the mental number line with a different approach (Liu et al., 2019). A previous work (Zorzi et al., 2002) had shown how neglect patients tend to displace the center of number intervals to the right. That is, when asked to tell the middle number between two numbers (1–5), neglect patients shift the center of the interval to the right (responding 4 instead of 3), in the same way as they do with a line bisection task. Using fMRI, Liu et al. (2019) applied the number bisection task and contrasted it with line bisection in healthy participants. The conjunction test for both tasks showed common activations mainly located bilaterally in parietal–frontal areas. They showed a bias towards the left hemisphere when contrasting number bisection minus physical bisection. This entailed a left hemisphere network: lingual gyrus, inferior frontal gyrus, precentral, inferior parietal lobule, the supplementary motor area, insula, caudate, inferior temporal gyrus and thalamus. The authors highlighted a pivotal role of the left supplementary motor area (SMA) which is relevantly coupled with the bilateral frontoparietal networks, especially during number bisection. Hence, this work unveiled bilaterality for number bisection. However, it stressed that that number bisection, more than line bisection, requires a left lateralized network.

### Neurostimulation studies of numerical processing in adults

While MRI was used in most studies, the electrophysiological responses were recorded in a few investigations conducted in epilepsy patients implanted with subdural electrodes. Thus Baek et al. (2018) found an increase in the high frequency broadband (HFB) activity in both the posterior inferior temporal gyrus and the intraparietal sulcus for equations presented in either digits or in number words. In the temporal gyrus the HFB activations were similar for digits and number words in the time of activation but differed in magnitude. In the intraparietal sulcus the activity was significantly delayed for number words in comparison to digits regardless of the hemisphere. A specific site in the posterior inferior temporal cortex was found by Pinheiro-Chagas et al. (2018) that activates during visual perception of numerals, with widespread adjacent responses when numerals are used in calculation. An initial burst of HFB activity decreased as the operands got larger. The authors concluded that while parietal sites appear to have a more sustained function in arithmetic computations, the inferior posterior inferior parietal area may contribute to early identification of the problem difficulty. These studies, however, do not seem to show any hemispheric asymmetry.

TMS has shown a role for both parietal lobes in processing quantity, including the left IPL (Sandrini et al., 2004). Not all results were consistent with fMRI studies (Salillas and Semenza, 2015). In a number comparison task between two-digit numbers and a reference, Göbel et al. (2001) found a stronger effect for closer distances larger than the references after left AG stimulation. In Andres et al. (2005) the stimulation of the posterior parietal cortices (PPC) impaired comparison for close numbers, while only left PPC stimulation disrupted the comparison between far numbers. Cappelletti et al. (2007) found that, while the left IPS was crucial for both symbolic with non-symbolic stimuli, facilitation appeared only with stimulation of the right IPS. Sasanguie et al. (2013) the other hand, showed the crucial role of the left IPS in the mapping between small symbolic and non-symbolic numerosities. Cohen Kadosh et al. (2012) highlighted a role of the right IPS in the facilitation effect during a numerical Stroop task for physical judgments. Grotheer et al. (2016) showed a causal involvement of the right temporo-occipital cortex in the processing of masked Arabic numbers.

Number magnitude tends to be contralaterally compatible with the MNL: right hemisphere with small numbers, and left hemisphere with big numbers. Cattaneo et al. (2009) found a variation in visual cortex excitability caused by the presentation of numbers: while small numbers increased the proportion of trials in which TMS induced phosphenes after right occipital stimulation, large numbers increased this proportion after left occipital stimulation. Rusconi et al. (2007) showed bilaterally a SNARC effect in the posterior parietal lobes. Later, Rusconi et al. (2011) underlined the importance of right IFG and the frontal eye fields in the SNARC effect. Finally, Salillas et al. (2009), see also Renzi et al. (2011) showed that the ventral IPS, in the occipital part of IPS and within the network of motion perception (Vanduffel et al., 2002; Orban et al., 2006), was causally linked to motion perception as well as to number comparison. A right hemisphere involvement was shown for small numbers, while large numbers seemed to be more bilateral. This motion-quantity association suggests that attention to motion may be a component of attention along the MNL (McCrink et al., 2007).

Most TMS studies on calculation showed lateralization effects: addition (Göbel et al., 2006; Salillas et al., 2012; Maurer et al., 2016; Montefinese et al., 2017), subtraction (Maurer et al., 2016; Montefinese et al., 2017; Fresnoza et al., 2020), multiplication (Andres et al., 2011; Salillas et al., 2012; Maurer et al., 2016; Fresnoza et al., 2020) and division (Maurer et al., 2016) were studied. Bilaterality was shown for subtraction but left lateralization for simple addition and multiplication.

Göbel et al. (2006) showed a role of the anterior and posterior left IPS for double digit addition, and no effect on the right hemisphere. Montefinese et al. (2017) however showed a stronger role of the right hIPS for addition, and a lesser asymmetry for the ventral IPS (vIPS). They showed, however, that bilateral hIPS and vIPS subserve addition and subtraction to different degrees. AG and supramarginal gyrus (SMG) bilaterally support addition and subtraction. The effects on the right SMG were stronger. ANG was more important for in addition, while SMG was more involved in subtraction. Fresnoza et al. (2020) distinguished retrieval from calculation strategies: the left AG was activated for both retrieval and calculation in multiplication, while it was only involved in retrieval during subtraction. No detrimental effect was found for left hIPS. Andres et al. (2011) had also contrasted subtraction and multiplication, showing a bilateral role of the hIPS for both operations. Salillas et al. (2012) explored addition and multiplication and found addition to be supported by the horizontal part of the IPS (hIPS) bilaterally, while multiplication was supported by the left hIPS and, bilaterally, by the ventral IPS (vIPS). Maurer et al. (2016) stimulated more than 50 brain sites evidencing the probability of error for addition, subtraction, multiplication and division. The higher error rates in addition appeared after inhibition of the right middle frontal gyrus, right AG, and superior temporal gyrus, as well as of the anterior superior temporal gyrus. In subtraction, the highest proportion of errors appeared after inhibition of the right AG, as well as of the right SMG and the middle frontal gyrus. In multiplication, errors were more frequent after inhibition of the left AG and of the middle frontal gyrus. Finally, in division the highest error rates were provoked by inhibition of the left inferior parietal lobe, the left middle frontal gyrus, the posterior superior temporal cortex bilaterally, and the right middle precentral gyrus.

## The developmental changes of laterality

The adult pattern of laterality is the result of a complex process in development. This section will summarize studies that concern the brain networks supporting numerical representations in the first stages of development, with a particular focus on lateralization and how it changes with neural maturation and enculturation. The differential role of the left IPS and the right IPS is evidenced through a meta-analysis at the end of this revision. Comprehensive and detailed reviews including other areas and functions (although not focused on lateralization) can be found in Arsalidou et al. (2018), Peters and De Smedt (2018), and Vogel and De Smedt (2021).

### Magnitude representation abilities

Behavioral studies (e.g., Izard et al., 2009; Benavides-Varela and Reoyo-Serrano, 2021) report that children are equipped with the ability to represent number nonverbally from an early age. Two basic abilities are distinguished when analyzing numerical capacities in development. The first ability, present since birth and independent from language, consists in encoding and processing non-symbolic numerical representations (Gelman and Butterworth, 2005; Dehaene, 2011). It implies distinguishing differences in the number of objects/dots in different sets. The second ability, at least partially independent from the first, consists of comprehending and using symbolic representation of numbers, for instance Arabic numbers and number words. Both abilities are gradually acquired and strongly rely on education and other capacities such as verbal counting or reading.

**Figure 1:**
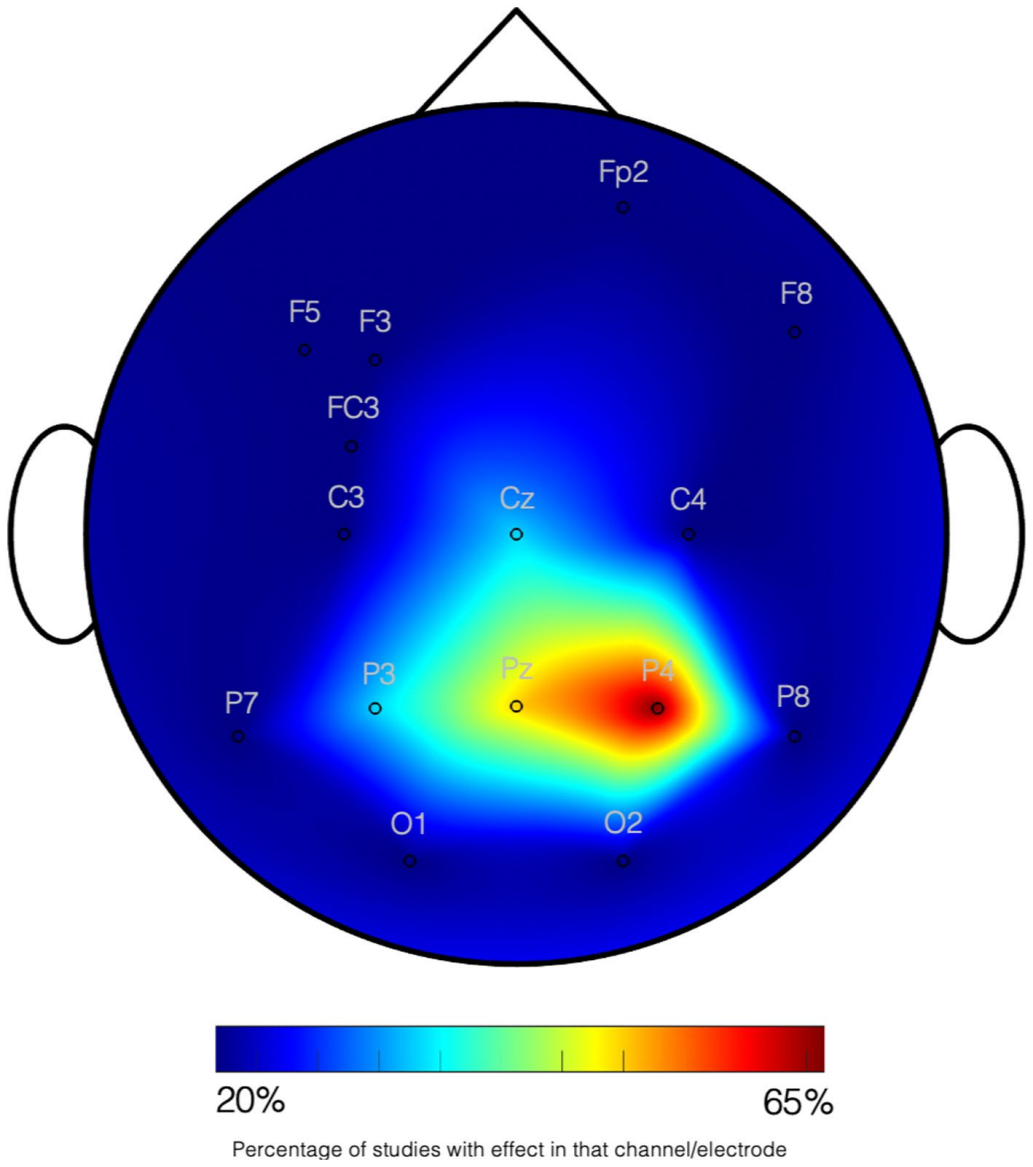
Graphical summary on studies on quantity processing in infants. Studies using EEG and fNIRS are joined according to the NIRS channels and EEG electrodes where effects have been found. All tasks imply numerosity processing. The scale indexes the percentage of studies (6 studies) showing an effect on the channel. All data collection systems have been transformed to the standard 10–20 system location. See Supplementary Table S1 for details on the studies.

Non-symbolic numerical abilities seem to be processed in the same areas in adults and young children. Clear asymmetries in the parietal regions are however evident in the first period of life. In fact, the left IPS does not have a strong involvement in sustaining basic numerical abilities early in life, while the right IPS readily encodes numerical magnitude independently of the amount of experience, mathematical knowledge, or age. For example, in the fMRI study of Cantlon et al. (2006), 4-year-old children and adults were exposed to a stream of dot arrays which occasionally changed in numerosity. Both groups showed stronger BOLD responses to novel than to familiar numerosities in the IPS. However, a stronger asymmetry towards the right hemisphere (i.e., resulting from lesser activity in the left IPS) was shown in children. Consistently, Holloway and Ansari (2010) tested older children (7- to 9-year-olds) and adults on both symbolic and non-symbolic number comparison. They showed that only a right-lateralized IPS activity was common to both groups. Studies with younger infants have also reported right lateralized brain responses to numerosity changes. For example, Izard et al. (2008) compared ERPs to changes in numerosity with those to changes in shape in 3-month-old infants. The response to numerical information originated primarily from right fronto-parietal regions. This study was consistent with an EEG study by Berger (2011) in 6–9-month-old infants, showing a right parietal activity related to the evaluation of numerosity in simple arithmetic equations. Likewise, in Libertus et al. (2009), parametric variations were found in neural oscillations as a function of numerical ratio in 7-month-old infants: alpha-band (6–8 Hz) oscillations over midline and right posterior scalp sites were found to be modulated by the ratio between familiar and novel numerosities Studies using fNIRS, also found that only the right parietal cortex of 6-month-olds was sensitive to numerosity (Hyde et al., 2010; Edwards et al., 2016). See Figure 1 for a graphical summary.

The lateralization of numerical processing changes throughout development with maturation and the exposure to education. Rivera et al. (2005) showed an age-related increment in the recruitment of the left SMG during a task with symbolic digits. No such increment was observed contralaterally. Likewise, Ansari and Dhital (2006) showed greater effects of numerical distance on the left IPS in adults with respect to children. Thus, the involvement of left IPS seems to develop as a function of age and of experience with Arabic numerals and number words (e.g., Ansari, 2008). Emerson and Cantlon (2015) found that neural responses in the left IPS change systematically as a function of children’s numerical discrimination acuity whereas right IPS responses are constant over a 1–2-year period in young children. The sustained activation of the right IPS and the incremental takeover of the left inferior parietal areas eventually change into a more bilateral pattern observed in adults (see above sections). Approximate judgments, for example, correlate with stronger activation in the right than in the left IPS, while exact judgments correlate with a larger activation in the left (Piazza et al., 2004, 2007).

Coordination between hemispheres may also gradually increase and sustain more efficient numerical processing throughout development. Cantlon et al. (2011) used fiber-based tractography to examine the relation between the integrity of the fibers of the corpus callosum connecting the left and right parietal lobes and performance on magnitude comparison tasks. Less integrity of these fibers was found in children compared with adults, with a correlation between integrity and the performance on the tasks. Thus, the interhemispheric connection seems to play an important role in the maturation of numerical representations. A later fMRI study by Park et al. (2014) showed that the degree of the effective connectivity between the right parietal and the left parietal cortex can predict individual scores in a test of mathematical achievement. In general, better white matter integrity in connections of the prefrontal cortex and the posterior parietal cortex or the occipitotemporal cortex coincides with better arithmetic performance (Matejko and Ansari, 2015).

Other non-parietal areas also change their pattern of lateralization during life. (Ansari et al., 2005) showed a right-lateralized frontal activity in a group of children while adults showed the typical bilateral IPS activation. This age-dependent frontal to parietal shift was replicated by Cantlon et al. (2009). Monitoring, the process of continuously evaluating the internal or external contingencies to optimize behavior, which is right-lateralized in the frontal lobe (Vallesi, 2012) could be working in children but gradually decrease in importance with age or practices.

### Arithmetic skills in typical development

Learning of arithmetic, the ability to add, subtract, multiply and divide symbolic whole numbers, has been shown to depend on a widespread set of interconnected brain areas which include the IPS as a key region, but also areas involved in domain-specific and domain-general processes such as language, working memory, long term memory and visuo-spatial abilities: the dorsolateral and ventrolateral prefrontal cortex, the posterior superior parietal lobule (PSPL), the fusiform gyrus, the angular gyrus, the supramarginal gyrus, and the hippocampus (Arsalidou et al., 2018). Activity associated with addition and subtraction increases over development in the right PSPL (Rosenberg-Lee et al., 2011a; Cho et al., 2012; Prado et al., 2014). Unlike for the IPS, the right PSPL is not considered to be specific to quantity-based processing, but to mechanisms of visuo-spatial attention, which might be progressively engaged or “recycled” in these tasks during arithmetic education (Hubbard et al., 2005; Dehaene and Cohen, 2007; Knops et al., 2009; Prado et al., 2014). Learning arithmetic facts in multiplications and additions, might rely on different strategies and developmental correlates (Suárez-Pellicioni et al., 2022). Interestingly, activation in language-related areas, is not consistently evidenced in developmental studies (e.g., Rosenberg-Lee et al., 2011a,b; Prado et al., 2014; Soltanlou et al., 2018; Declercq et al., 2022). Learning arithmetical facts in school-age children have been correlated with activity in the left-middle temporal gyrus (Prado et al., 2014) and more consistently with the hippocampus (Rivera et al., 2005; De Smedt et al., 2011). Reduced brain activity in the prefrontal cortex and increased activity in the right hippocampus were shown with the shift from the use of counting to memory-based strategies (Qin et al., 2014). Cho et al. (2012) found that children who frequently used a retrieval strategy exhibited enhanced activity and connectivity from the right hippocampus and other areas in the frontal cortex, when compared to children who used procedural strategies. De Smedt et al. (2011) reported that the left hippocampus showed increased brain activity during fact retrieval problems in 10-to-12-year-olds (De Smedt et al., 2011). Since adults seem to rely mainly on lower parietal areas, it has been suggested that the role of the hippocampus might be to encode new arithmetic facts before their representations can be transferred to language-related regions (De Smedt et al., 2011; Peters and De Smedt, 2018). The lateralization of the response in the hippocampus, however, has not been clarified in the literature.

### Arithmetic skills in developmental dyscalculia

One last issue connected with laterality concerns developmental dyscalculia (DD). Differences between typically developing (TD) children and children with DD have been reported in brain areas linked to general learning skills, including the hippocampal circuitry, the superior parietal lobule (SPL) and the prefrontal cortex (Rotzer et al., 2008; Rykhlevskaia et al., 2009). Differences have also been found in the right IPS (Molko et al., 2003; Rotzer et al., 2008), the left IPS (Isaacs et al., 2001) or bilaterally (Rykhlevskaia et al., 2009). A reduced white matter integrity of the superior longitudinal fasciculus is reported for DD (Rykhlevskaia et al., 2009). These tracts appeared to increase with age in the TD but not in the DD children (Ranpura et al., 2013). No clear differences between the anatomical alterations in the left and the right hemispheres emerge from these studies.

Brain functional alterations have also been reported in DD. Kucian et al. (2006) found that, when solving an approximate arithmetic task, children with DD, compared to TD, had weaker activation in the left IPS as well as right inferior frontal gyrus and right medial frontal gyrus. Price et al. (2007) found that children with DD showed significantly less brain activity in the right IPS compared to age- and IQ-matched TD children during a non-symbolic magnitude comparison task. Right or bilateral IPS differences compared to controls were also observed in further symbolic comparison tasks (Soltész et al., 2007; Mussolin et al., 2010). In contrast, Michels et al. (2018) revealed increased activation in the left IPS, frontal cortex, and visual areas in DD during number processing, as well as functional hyperconnectivity in parietal, frontal, visual, and temporal regions. This finding has been attributed to compensatory mechanisms and the increment of cognitive control resources in DD (Kaufmann et al., 2009). Children with DD, differently from TD children, recruit the fronto-parietal network to the same extent for both easy and hard problems (De Smedt et al., 2011; Ashkenazi et al., 2012). A recent meta-analysis by Tablante et al. (2023) considered the findings of 24 studies with 728 children, revealed that the most consistent dysfunction across studies was in the right parietal lobe along the intraparietal sulcus (IPS), suggesting a core deficit in quantity processing.

## Laterality patterns of neuroimaging studies in children

In this section we systematically assess the degree of lateralization of brain areas associated with number comprehension and in simple calculation (addition) in children. There are recent meta-analyses focusing on this issue (Arsalidou et al., 2018; Peters and De Smedt, 2018), yet concrete measures of lateralization across studies were not included. Based on the literature compiled by those meta-analysis, homogeneity in the tasks and the concrete dependent measures of the studies is emphasized. First, the literature on quantity was split in two subsets, attending to the use of symbolic or non-symbolic material. Then, laterality indexes (LIs) were obtained from each of the two subsets. Second, the literature on calculation was studied for lateralization too. Because studies focusing on subtraction and multiplication are sparse, they did not provide enough statistical power for the analyses. Thus, only addition problems were analyzed. To achieve further homogeneity, only studies that involved neuroimaging (fMRI) on school-age children over 6 years were included. fMRI studies with younger children were excluded because they are scarce (Cantlon et al., 2006; Park et al., 2014; Kersey and Cantlon, 2017). All correlational analysis with other variables were not considered, given the variability in measures across studies. See Supplementary material for details on the included studies.

Within the *non-symbolic studies subset*, all the tasks involved quantity comparison (Ansari and Dhital, 2006; Kaufmann et al., 2008; Kucian et al., 2011; Berteletti et al., 2014, 2015; Demir-Lira et al., 2016). The attended contrast was ratio or distance in all of them. The average age between studies was 10.67 y.o. (SD: 1.09) and the total number of participants was 128.Within the *symbolic subset of studies*, the tasks involved the comparison of two Arabic digits (Ansari et al., 2005; Meintjes et al., 2010; Bugden et al., 2012; Gullick and Wolford, 2013), the matching between symbolic and non-symbolic formats (Emerson and Cantlon, 2012, 2015), and the adaptation to symbolic numerical stimuli (Vogel et al., 2015). The attended contrast were ratio or distance effects (Ansari et al., 2005; Meintjes et al., 2010; Bugden et al., 2012; Gullick and Wolford, 2013; Vogel et al., 2015) or a contrast to non-numerical stimuli (Meintjes et al., 2010; Emerson and Cantlon, 2012, 2015). Differing from Arsalidou et al. (2018), and again with the main goal of increasing homogeneity across studies, the contrasts for negative numbers in Gullick and Wolford (2013) were not included. The average age between studies was 9.77 y.o. (SD: 1.84) and the total number of participants was 136.

Within the *addition studies*, all except one study involving passive mental calculation (Kawashima et al., 2004) involved a verification task with one solution (Rosenberg-Lee et al., 2011a; Ashkenazi et al., 2012; Cho et al., 2012; Metcalfe et al., 2013; Qin et al., 2014), a selection between two (Meintjes et al., 2010; De Smedt et al., 2011) or three solutions (Davis et al., 2009). Most of the used contrasts involved one-digit operands that were contrasted to the sum +1 (Rosenberg-Lee et al., 2011a; Ashkenazi et al., 2012; Cho et al., 2012; Metcalfe et al., 2013; Qin et al., 2014). Meintjes et al. (2010) and Davis et al. (2009) implied the contrast to equivalent non-numeric symbolic tasks. The contrast from De Smedt et al. (2011) involved addition > subtraction, and Kawashima et al. (2004) involved the contrast addition > fixation. The average age between studies was 9.06 y.o. (SD: 1.57) and the total number of participants was 136. Supplementary Table S2 for details the included studies in each of the three sets.

**Figure 2:**
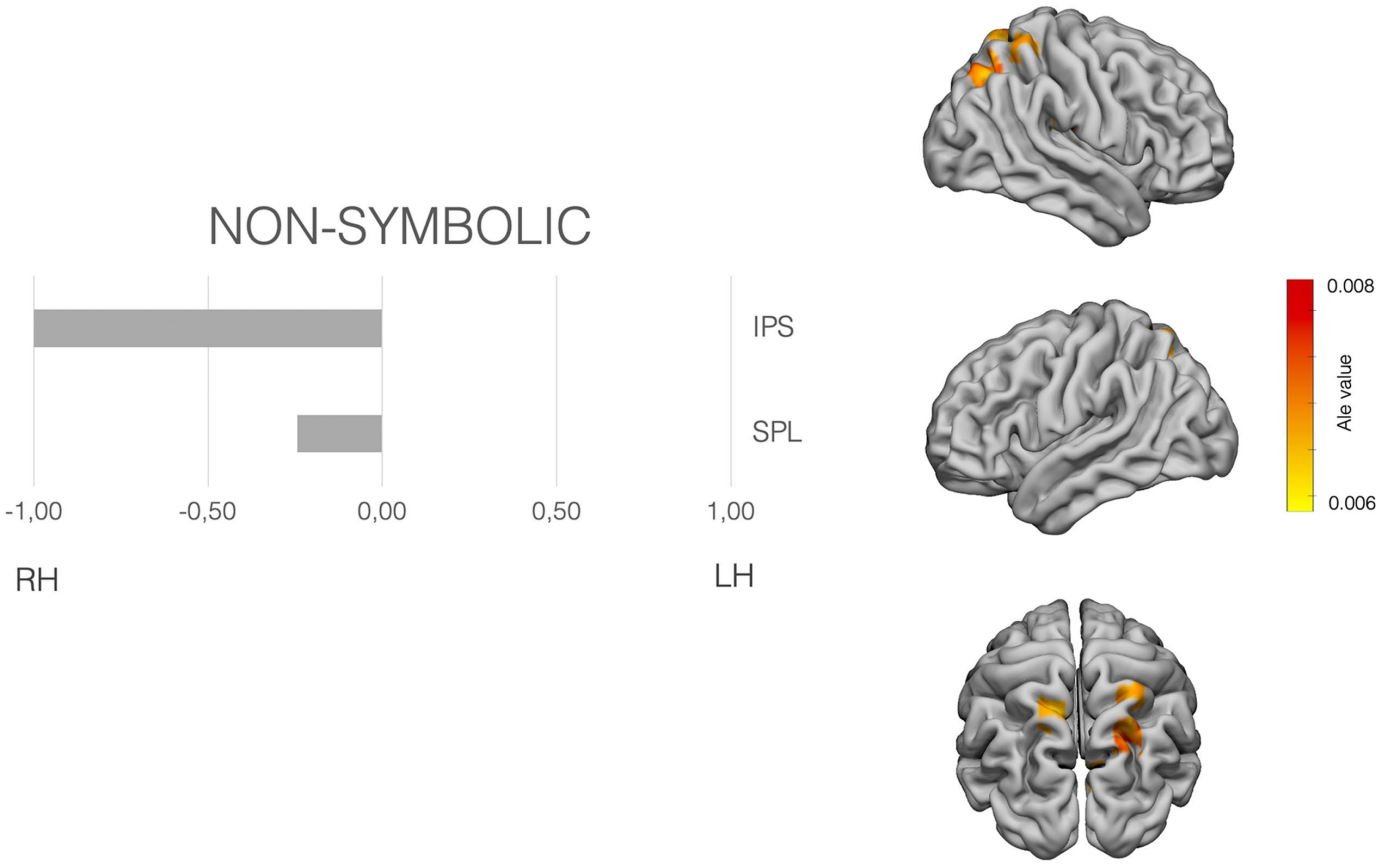
Laterality pattern for non-symbolic quantity processing in school age children. Right: Results of meta-analysis displaying supra-threshold ALE values (corrected *p* value < 0.001). Left: LIs values computed on those ALE values. Range −1 to +1. Negative values mean right hemisphere (RH) predominance. Positive means left hemisphere (LH) predominance. Age range: 8.6–11.5 y.o. See Supplementary Table S3 for details on the suprathreshold clusters.

**Figure 3:**
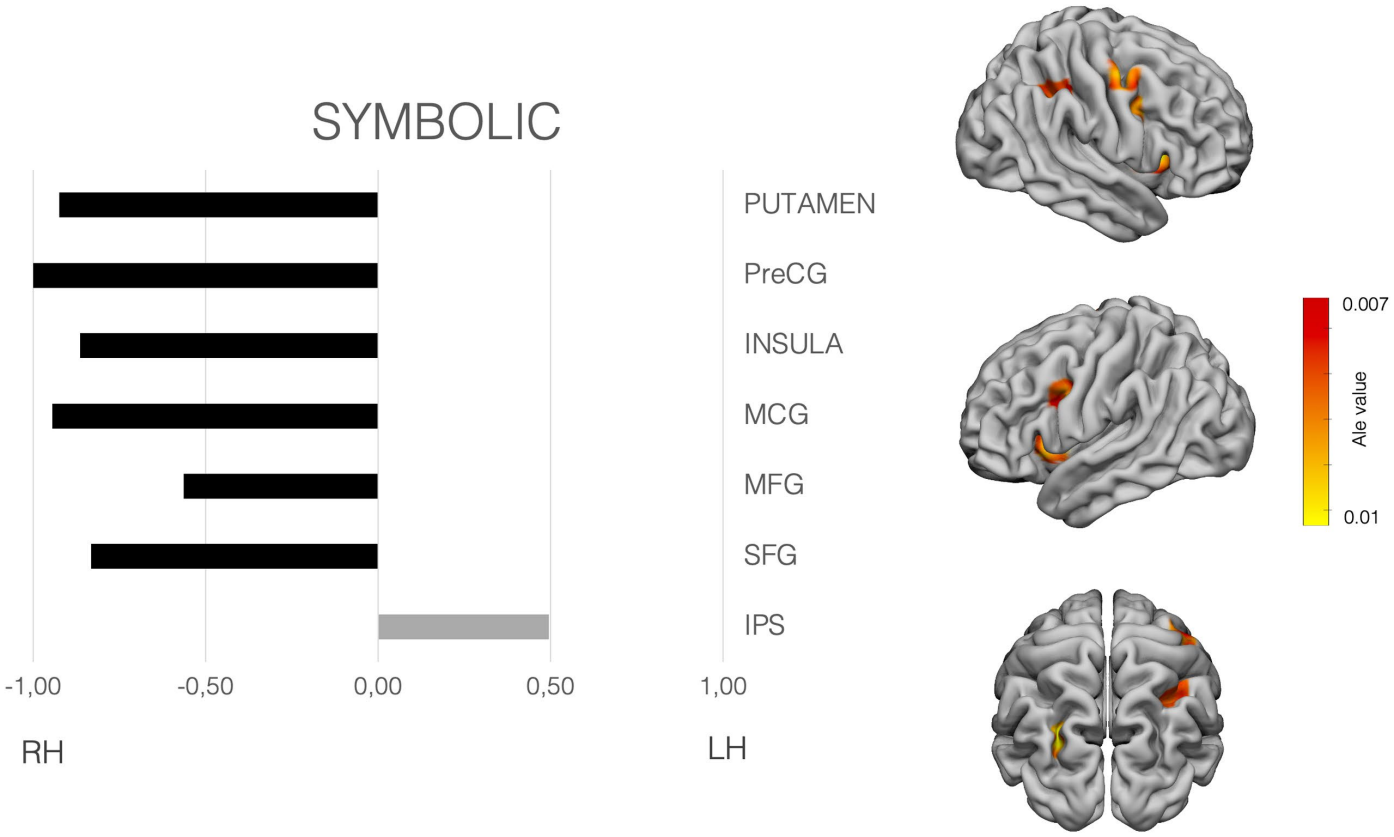
Laterality pattern for symbolic quantity processing in school age children. Right: Results of meta-analysis displaying supra-threshold ALE values (corrected *p* value < 0.001). Left: LIs values computed on those ALE values. Range −1 to +1. Negative values mean right hemisphere (RH) predominance. Positive means left hemisphere (LH) predominance. Age range: 6.6–12.7 y.o. See Supplementary Table S3 for details on the suprathreshold clusters.

**Figure 4:**
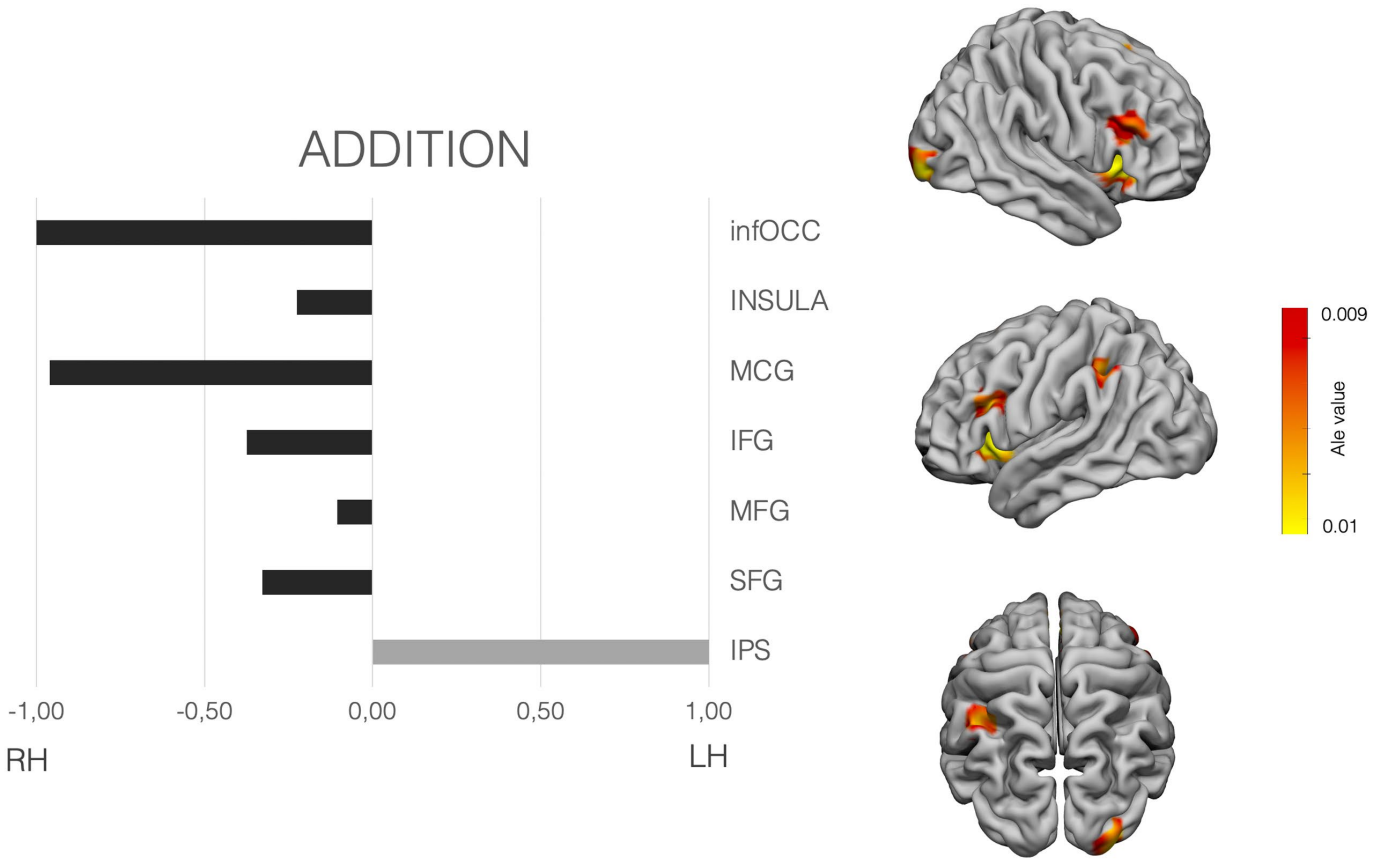
Laterality pattern for simple addition in school age children. Right: Results of meta-analysis displaying supra-threshold ALE values (corrected *p* value < 0.001). Left: LIs values computed on those ALE values. Range −1 to +1. Negative values mean right hemisphere (RH) predominance. Positive means left hemisphere (LH) predominance. Age range: 7.7–11.9 y.o. See Supplementary Table S3 for details on the suprathreshold clusters.

The meta-analysis was performed with Ginger-Ale (http://brainmap.org/ale/; Research Imaging Center of the University of Texas in San Antonio), which estimates the likelihood that at least one of the coordinates falls in the template stereotaxic space, in *activation likelihood estimated* (ALE) values. All the coordinates were transformed to the MNI space, considering the method used by the authors for normalization. Then the meta-analysis was conducted in the MNI space. ALE maps were calculated with a corrected value of *p* < 0.001. Figures 2–4 shows the ALE maps for the non-symbolic, symbolic subsets, and for addition, respectively.

To examine the hemispheric asymmetries, the MATLAB script AveLI (Matsuo et al., 2012; http://aveli.web.fc2.com) was used. This program considers not only the extension in voxels falling within two masks (left and right hemisphere mask), but also the intensity of the ALE value in our case. The masks used were selected after observing the areas with suprathreshold in each of the ALE maps (see Figures 2–4 and Supplementary material). The masks were derived from a Freesurfer atlas. Then, the three suprathreshold ALE values maps (symbolic, non-symbolic and addition) were entered in the analysis, considering each of the selected masks.

The non-symbolic quantity subset analysis showed suprathreshold ALE values in the bilateral superior parietal lobe and the right IPS. The laterality indexes show a clear right asymmetry in the IPS, the involvement of bilateral SPLs (see Figure 2).

symbolic quantity subset analyses showed significant clusters in the bilateral IPS, middle frontal gyrus (MFG), superior frontal gyrus (SFG). Moreover, the putamen, claustrum, the insular cortices and precentral SFG, MFG, Middle Cingulate cortex, showed also significant ALE values. Attending to the LIs, and clearly differing from the non-symbolic subset, the IPS showed a left hemisphere preponderance. All the rest of areas showed a dominance for the right hemisphere (see Figure 3).

The data on quantity processing confirm that the parietal areas used by children for processing numerical quantity differ in lateralization for symbolic and non-symbolic stimuli, especially for the IPS: the left IPS is driving the process with symbolic numbers while the right IPS drives quantity processing when the stimuli are non-symbolic. Moreover, the processing of symbolic stimuli involves of a wider cortical network that is bilateral, but with a right hemisphere dominance. This wider network involves a higher processing weight of the right putamen, insula, middle cingulate gyrus, precentral gyrus, MFG, and SFG, which seem not necessary for the non-symbolic material. Supplementary Figure S1 shows the analysis performed including also studies on children from 4 years. Frontal areas loose relevance in the non-symbolic subset with age. Yet insula and putamen are still irrelevant areas on non-symbolic quantity processing in younger children.

As per exact calculation (addition), the ALE-maps show suprathreshold values in the right occipital pole, bilateral insula and IFG, bilateral MFG, the right middle cingulate cortex, the left SMA and the left parietal cortex only. LIs shows a clear left dominance for the IPS. And a tendency for bilaterality, yet with slighter bigger dominance for the right frontal hemisphere (IFG, MFG and SFG), as well as bilateral insular cortex, with a similar slight right predominance (see Figure 4).

To summarize, the results indicate that, at school age, the laterality pattern changes as a function of the numerical stimuli presented. The use of non-symbolic stimuli in magnitude comparison tasks entails a clear involvement of the parietal areas in the right hemisphere. Tasks with number symbols instead, shift the laterality pattern with significantly stronger activation in the left IPS. This pattern resembles the one recently reported by Escobar-Magariño et al. (2022) in adults. It is also somehow in agreement with the proposal of Cohen Kadosh et al. (2007) that parietal areas would process magnitude in a format dependent manner. Thus, the overall right lateralization in children reported in previous meta-analyses (e.g., Arsalidou et al., 2018) have been influenced by the non-symbolic literature. Non-symbolic quantity processing in school-age overall seems to be already rely mainly on the parietal cortex.

Besides parietal areas, symbolic quantity processing and additions in children rely on more extended brain activity. Areas in the frontal, insular and occipital cortex are also recruited in these tasks and might be related with effortful control, learning and generally less automatized processing mechanisms compared to adults (see also Arsalidou and Taylor, 2011; Arsalidou et al., 2018) These areas show clear lateralization to the right hemisphere. Notably, children show a more extended and less specialized network.

Finally, in processing symbolic stimuli, the insular cortex and the cingulate are active during both quantity and addition processes. This agrees with Arsalidou and Taylor (2011) and Arsalidou et al. (2018), however these areas seem to play a role only with symbolic stimuli. More studies should address the exact function of these areas both in calculation and numerical quantity processing.

## Conclusion

Lateralization of math in the brain has been investigated in several ways and considerable progress has been made since Sperry’s times. Besides clinical observations, still very important in several respects, new methodologies, unknown before the end of the last century, have been employed.

The notion emerges of an interaction of the two hemispheres involving complex brain networks. However, each hemisphere seems to maintain its own role. An important, perhaps the newest, result is that there is more than a simple difference along the verbal/spatial dimensions distinguishing the work of each hemisphere. Very specific arithmetical functions like remembering the spatial templates for complex operations, or processing of zero in complex numbers, are indeed sustained in specific right sided areas.

Importantly, the numerical brain undergoes a complex pattern of development. The ability to represent numbers nonverbally is supported mainly by right IPS and seems to be present from a very early age. This area is shown to be dysfunctional in developmental acalculia. Growth and education then shape less mature networks into more efficient ones. Later, at school ages, with the exposure to numerical codes like the Arabic code and written number words, a more extended set of brain areas appears involved, while the role of the left IPS becomes dominant.

## Author contributions

ES: Conceptualization, Formal analysis, Methodology, Writing – original draft, Writing – review & editing. SB-V: Conceptualization, Funding acquisition, Validation, Writing – original draft, Writing – review & editing. CS: Conceptualization, Validation, Writing – original draft, Writing – review & editing.
