# Supplementary material for: The brain lateralization and development of math functions: progress since Sperry, 1974
Source: Front Hum Neurosci. 2023 Oct 27;17:1288154. doi: 10.3389/fnhum.2023.1288154 (PMC10641455; doi:10.3389/fnhum.2023.1288154)
Supplement: Supplementary file 1 [file Data_Sheet_1.PDF]

TABLE S1 – ELECTRODES/CHANNELS WITH EFFECTS IN EACH STUDY.

	Izard (2008)	Libertus (2009)	Hyde (2010)	Edwards (2016)	Libertus (2011)	Hyde (2011)		
**Age:**	3.4m (3-4.1 m)	7m3d (6m10d-7m27d)	5.5m-6.5m	6.64m(SD 0.62)	7m5d(17d)	6-7.5m		
**Technique:**	EEG	EEG	fNIRS	fNIRS	EEG	EEG		
**Dependent measure:**	Number Deviant vs Standard	Ratio	Number Deviant vs Standard	Number change vs. no change	Ratio	Small vs Large Numerosity change		
**Channel LIST**							**Value (over 6 studies)**	**%**
C3	C3						1	16,7
C4	C4						1	16,7
P4	P4		P4	P4		P4	4	66,7
P3	P3					P3	2	33,3
Cz	Cz	Cz					2	33,3
Pz	Pz	Pz			Pz		3	50,0
AF4							1	16,7
F4							1	16,7
F5							1	16,7
F3							1	16,7
FC3							1	16,7
P7						P7	1	16,7
P8						P8	1	16,7
O1						O1	1	16,7
O2						O2	1	16,7

When the electrodes were in a different location system (i.e., EGI system) they were replaced for the equivalent 10-20 positions.
Scalp plot in Figure 1 was computed from the percentages above the total number of studies.

TABLE S2 - DETAILS OF THE STUDIES INCLUDED IN THE ANALYSIS.

NON-SYMBOLIC

first author	year	n	age	task specifics	BOLD Dependent variable	foci
Ansari	2006	9	10.4	non-symbolic comparison	distance	3
Berteletti	2015	39	11.33(1.5)	non-symbolic comparison (localizer)	ratio	2
Berteletti	2014	20	11.5(1.58)	non-symbolic comparison (localizer)	ratio	2
Demir-Lira	2016	33	10.9(1.5)	non-symbolic comparison (localizer)	ratio	3
Kucian	2011	15	11.3(1.3)	non-symbolic comparison	ratio	14
Kaufmann	2008	12	8.6(1.2)	comparison between finger number	finger comparison > rest	6
		128				**30**

SYMBOLIC

first author	year	n	age	task specifics	BOLD Dependent variable	foci
Ansari	2005	12	10.4(9.2-11.11)	Symbolic comparison	distance	9
Budgen	2012	17	8.8(0.73)	Symbolic comparison	ratio	6
Emerson	2015	17	8.24(2.26)	Match symbol - non-symbol	numerical > non numerical	5
Meintjes	2010	16	10.5(1.2)	Symbolic comparison - two choices	numberic > non numeric symbols	17
Emerson	2012	24	6.63(1.4)	Match symbol - non-symbol	numerical > non numerical	5
Vogel	2015	19	10.2(2.55)	Adaptation to symbolic quantity	ratio	5
Gullick(a)	2013	16	10.7(9:11-11:9)	Symbolic comparison	distance	9
Gulllick(b)	2013	15	12.7(11:9-13:5)	Symbolic comparison	distance	12
						**68**

ADDITION

first author	year	n	mean age	task specifics	BOLD Dependent variable	foci
Ashkenazi	2012	17	8.12 (0,5)	verification (operands 2 to 9)	one digit vs (+1).	23
Metcalfe	2013	74	7.8 (0.7)	verification (operands 2 to 9)	one digit vs (+1).	6
Qin	2014	28	8.26 (0.53)//9.45(0.88)	verification (operands 2 to 9)	one digit vs (+1) (T1+T2) (supplementary material)	17
Rosenberg-lee	2011	90	7.67(0.40)//8.67(0.40)	verification (operands 2 to 9)	one digit vs (+1) (ages collapseed)	5
Cho	2012	86	7.7	verification (operands 2 to 9)	one digit vs (+1) (supplementary material)	15
Davis	2009	27	8.1(0.4)	Exact - multiple choice (3 options)	one digit three choices vs. symbols three choices	5
De Smedt	2011	18	11.88(0.89)	verification (operands 2 to 9) - multiple choice (x2)	addition>subtraction main effect (no interact by group) choice between two answers	1
Meintjes	2010	16	10.5(1.2)	verification (operands 2 to 9) - multiple choice (x2)	addition > control (symbols)	25
Kawashima	2004	8	11.6(1.6)	mental calculation	addition > fixation (supplementary material)	6
		**364**				**103**

TABLE S3 – SUPRATHRESHOLD CLUSTERS FROM ALE ANALISIS

NON-SYMBOLIC

cluster#	Hemisphere	Location	Area	BA	voxels	x	y	z	ALE
1	RH	Occipital	cuneus	17	19	14	-78	6	0,009
2	RH	Parietal	precuneus	31,18	19	12	-74	22	0,010
3	RH	Parietal	IPS/SPL	7	48	26	-68	46	0,009
4	LH	Parietal	SPL	7	19	-16	-60	58	0,010
5	RH	Parietal	SPL	7;5;40	19	28	-48	62	0,009

IPS = Intraparietal Sulcus; SPL = Superior Parietal Lobe

SYMBOLIC

cluster#	Hemisphere	Location	Area	BA	voxels	x	y	z	ALE
1	LH	Insula	Insula	13;47	71	-32	14	0	0,015
2	RH	subcortical, insula	Putamen, insula		253	32	12	6	0,018
3	LH	Parietal	IPL, ANG	39	95	-28	-66	28	0,016
4	RH	Frontal	Precentral, IFG		54	34	0	34	0,013
5	LH	Frontal	Precentral, IFG	9	40	-42	6	34	0,010
6	RH	Parietal	IPL, SMG	40	54	36	-42	40	0,010
7	RH	Cingulate Gyrus	Cingulate Mid (SMA)	32	82	4	12	44	0,013
8	RH	Frontal	MFG	6	44	45	-3	59	0,013

ANG= Angular Gyrus; IPL= Inferior Parietal Lobe; IFG= Inferior Frontal Gyrus; MFG= Middle Frontal Gyrus; SMG= Supramarginal Gyrus; SMA= Supplementary Motor Area

ADDITION

cluster#	Hemisphere	Location	Area	BA	voxels	x	y	z	ALE
1	RH	Occipital	Occipital pole	18	61	26	-94	-4	0,018
2	RH	Insula, Frontal	Insula, IFG	13;47	253	34	22	0	0,030
3	LH	Insula, Frontal	Insula, IFG	13;47	173	-30	20	2	0,034
4	LH	Frontal	IFG (Pars Triangularis)	45	61	-42	21	23	0,014
5	RH	Frontal	MFG	46	61	52	30	26	0,014
6	LH	Cingulate gyrus	Middle Cingulate	32	15	-8	18	34	0,010
7	RH	Frontal	Medial FG	6;32	80	4	26	40	0,017
8	LH	Parietal	IPS; SMG	40	50	-44	-46	46	0,016
9	LH	Frontal	SMA	6;8	82	0	16	52	0,019

FG= Frontal Gyrus

FIGURE S1 – ALE ANALYSIS INCLUDING CHILDREN BETWEEN 4 AND 6 Y.O.

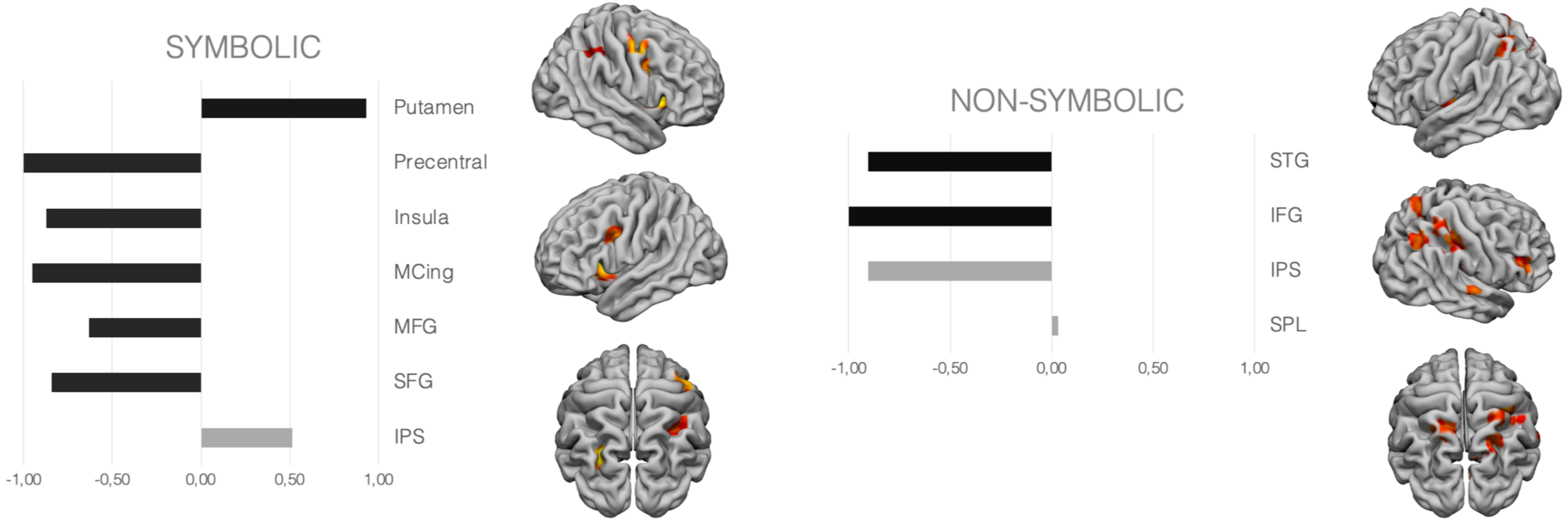

This analysis includes Cantlon et al., (2006); Kersey et al. (2017) for non-symbolic tasks, and Park et al (2013) for symbolic tasks.

SUPPLEMENTAL REFERENCES

Libertus, M. E., Brannon, E. M., and Woldorff, M. G. (2011). Parallels in stimulus-driven oscillatory brain responses to numerosity changes in adults and seven-month-old infants. Dev. Neuropsychol. 36, 651–667. doi:10.1080/87565641.2010.549883.

Hyde, D. C., and Spelke, E. S. (2011). Neural signatures of number processing in human infants: Evidence for two core systems underlying numerical cognition. Dev. Sci. 14, 360–371. doi:10.1111/j.1467-7687.2010.00987.x.
